# Provisional Mortality Data — United States, 2020

**DOI:** 10.15585/mmwr.mm7014e1

**Published:** 2021-04-09

**Authors:** Farida B. Ahmad, Jodi A. Cisewski, Arialdi Miniño, Robert N. Anderson

**Affiliations:** 1 National Center for Health Statistics, CDC.

CDC’s National Vital Statistics System (NVSS) collects and reports annual mortality statistics using data from U.S. death certificates. Because of the time needed to investigate certain causes of death and to process and review data, final annual mortality data for a given year are typically released 11 months after the end of the calendar year. Daily totals reported by CDC COVID-19 case surveillance are timely but can underestimate numbers of deaths because of incomplete or delayed reporting. As a result of improvements in timeliness and the pressing need for updated, quality data during the global COVID-19 pandemic, NVSS expanded provisional data releases to produce near real-time U.S. mortality data.* This report presents an overview of provisional U.S. mortality data for 2020, including the first ranking of leading causes of death. In 2020, approximately 3,358,814 deaths^†^ occurred in the United States. From 2019 to 2020, the estimated age-adjusted death rate increased by 15.9%, from 715.2 to 828.7 deaths per 100,000 population. COVID-19 was reported as the underlying cause of death or a contributing cause of death for an estimated 377,883 (11.3%) of those deaths (91.5 deaths per 100,000). The highest age-adjusted death rates by age, race/ethnicity, and sex occurred among adults aged ≥85 years, non-Hispanic Black or African American (Black) and non-Hispanic American Indian or Alaska Native (AI/AN) persons, and males. COVID-19 death rates were highest among adults aged ≥85 years, AI/AN and Hispanic persons, and males. COVID-19 was the third leading cause of death in 2020, after heart disease and cancer. Provisional death estimates provide an early indication of shifts in mortality trends and can guide public health policies and interventions aimed at reducing numbers of deaths that are directly or indirectly associated with the COVID-19 pandemic.

CDC analyzed provisional NVSS death certificate data for deaths occurring among U.S. residents in the United States during January–December 2020. The numbers and rates of overall deaths and COVID-19 deaths were assessed by age, sex, and race/ethnicity (categorized as Hispanic, non-Hispanic White [White], Black, non-Hispanic Asian, non-Hispanic AI/AN, non-Hispanic Native Hawaiian or other Pacific Islander [NH/PI], non-Hispanic multiracial, and unknown). Causes of death were coded according to the *International Classification of Diseases, Tenth Revision* (ICD-10), which describes disease classification and the designation of underlying cause of death (*1*,*2*). Numbers and rates of COVID-19 deaths include deaths for which COVID-19 was listed on the death certificate as a confirmed or presumed underlying cause of death or contributing cause of death (ICD-10 code U07.1). COVID-19 was the underlying cause of approximately 91% (345,323) of COVID-19–associated deaths during 2020 (*3*). Leading underlying causes of death were calculated and ranked (*4*). Deaths that occurred in the United States among residents of U.S. territories and foreign countries were excluded.^§^ Age was unknown for 86 (<0.01%) decedents, and race/ethnicity was unknown for 9,135 (0.27%). There were no records with unknown sex. To describe the trend in deaths during 2020, the number of deaths from all causes and from COVID-19 were calculated for each week. Midyear U.S. Census Bureau population estimates (July 1, 2020) were used to calculate estimated death rates per 100,000 standard population (*5*). Age-adjusted death rates were calculated for deaths by sex and race/ethnicity, and crude death rates were calculated by age. Age-adjusted death rates for 2020 were also compared with those from 2019 (*6*).

In 2020, approximately 3,358,814 deaths occurred in the United States (Table). The age-adjusted rate was 828.7 deaths per 100,000 population, an increase of 15.9% from 715.2 in 2019. The highest overall numbers of deaths occurred during the weeks ending April 11, 2020, (78,917) and December 26, 2020 (80,656) (Figure 1). Death rates were lowest among persons aged 5–14 years (13.6) and highest among persons aged ≥85 years (15,007.4); age-adjusted death rates were higher among males (990.5) than among females (689.2).

**TABLE:** Provisional* number and rate of total deaths and COVID-19–related deaths, by demographic characteristics — National Vital Statistics System, United States, 2020

Characteristic	No. (rate)^†^	
	Total deaths	COVID-19 deaths^§^
**Total**	**3,358,814 (828.7)**	**377,883 (91.5)**
**Age group, yrs**		
<1	19,146 (506.0)	43 (1.1)
1–4	3,469 (22.2)	24 (0.2)
5–14	5,556 (13.6)	67 (0.2)
15–24	35,470 (83.2)	587 (1.4)
25–34	72,678 (157.9)	2,527 (5.5)
35–44	103,389 (246.2)	6,617 (15.8)
45–54	189,397 (467.8)	17,905 (44.2)
55–64	436,886 (1,028.5)	44,631 (105.1)
65–74	669,316 (2,068.8)	80,617 (249.2)
75–84	816,307 (4,980.2)	104,212 (635.8)
≥85	1,007,114 (15,007.4)	120,648 (1,797.8)
Unknown	86 (—)	5 (—)
**Sex**		
Female	1,602,366 (689.2)	172,689 (72.5)
Male	1,756,448 (990.5)	205,194 (115.0)
**Race/Ethnicity**		
Hispanic	304,488 (724.1)	68,469 (164.3)
White, non-Hispanic	2,467,419 (827.1)	228,328 (72.5)
Black, non-Hispanic	443,116 (1,105.3)	59,871 (151.1)
Asian, non-Hispanic	90,519 (457.9)	13,334 (66.7)
American Indian or Alaska Native, non-Hispanic	24,279 (1,024.0)	4,504 (187.8)
Native Hawaiian or other Pacific Islander, non-Hispanic	4,424 (828.4)	679 (122.3)
Multiracial, non-Hispanic	15,434 (378.8)	1,125 (31.8)
Unknown	9,135 (—)	1,573 (—)

* National Vital Statistics System provisional data are incomplete. Data from December are less complete due to reporting lags. These data exclude deaths that occurred in the United States among residents of U.S. territories and foreign countries.

^†^ Deaths per 100,000 standard population. Age-adjusted death rates are provided by sex and race/ethnicity.

^§^ Deaths with confirmed or presumed COVID-19 as an underlying or contributing cause of death, with *International Classification of Diseases, Tenth Revision* code U07.1.

**FIGURE 1:**
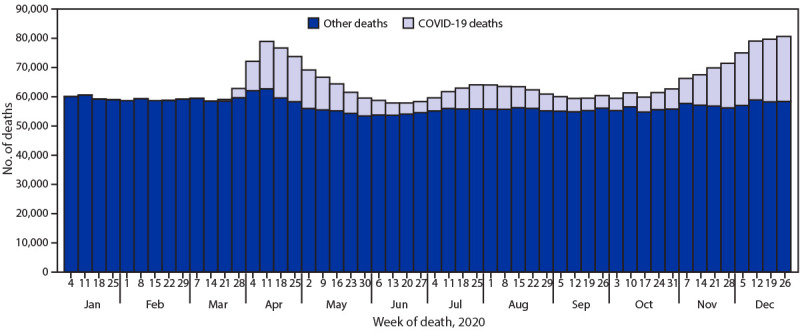
Provisional* number of COVID-19–related deaths^†^ and other deaths, by week — National Vital Statistics System, United States, 2020 * National Vital Statistics System provisional data are incomplete. Data from December are less complete due to reporting lags. Deaths that occurred in the United States among residents of U.S. territories and foreign countries were excluded. ^†^ Deaths with confirmed or presumed COVID-19 as an underlying or contributing cause of death, with *International Classification of Diseases, Tenth Revision* code U07.1.

During 2020, COVID-19 was listed as the underlying or contributing cause of 377,883 deaths (91.5 per 100,000 population). COVID-19 death rates were lowest among children aged 1–4 years (0.2) and 5–14 years (0.2) and highest among those aged ≥85 years (1,797.8). Similar to the rate of overall deaths, the age-adjusted COVID-19–associated death rate among males (115.0) was higher than that among females (72.5).

Age-adjusted death rates differed by race/ethnicity. Overall age-adjusted death rates were lowest among Asian (457.9 per 100,000 population) and Hispanic persons (724.1) and highest among Black (1,105.3) and AI/AN persons (1,024.0). COVID-19–associated death rates were lowest among multiracial (31.8) and Asian persons (66.7) and highest among AI/AN (187.8) and Hispanic persons (164.3). COVID-19 was listed as the underlying cause of 345,323 deaths during 2020 and was the third leading underlying cause of death, after heart disease (690,882 deaths) and cancer (598,932) (Figure 2).

**FIGURE 2:**
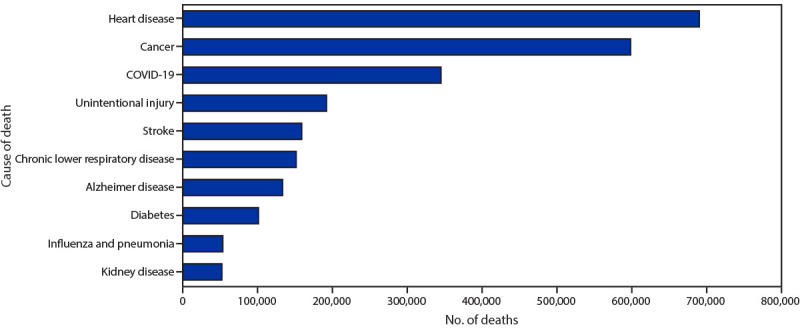
Provisional* number of leading underlying causes of death^†^ — National Vital Statistics System, United States, 2020 * National Vital Statistics System provisional data are incomplete. Data from December are less complete due to reporting lags. Deaths that occurred in the United States among residents of U.S. territories and foreign countries were excluded. ^†^ Deaths for which COVID-19 was a contributing, but not the underlying, cause of death are not included in this figure.

## Discussion

During January–December 2020, the estimated 2020 age-adjusted death rate increased for the first time since 2017, with an increase of 15.9% compared with 2019, from 715.2 to 828.7 deaths per 100,000 population. COVID-19 was the underlying or a contributing cause of 377,883 deaths (91.5 deaths per 100,000). COVID-19 death rates were highest among males, older adults, and AI/AN and Hispanic persons. The highest numbers of overall deaths and COVID-19 deaths occurred during April and December. COVID-19 was the third leading underlying cause of death in 2020, replacing suicide as one of the top 10 leading causes of death (*6*).

The findings in this report are subject to at least four limitations. First, data are provisional, and numbers and rates might change as additional information is received. Second, timeliness of death certificate submission can vary by jurisdiction. As a result, the national distribution of deaths might be affected by the distribution of deaths from jurisdictions reporting later, which might differ from those in the United States overall. Third, certain categories of race (i.e., AI/AN and Asian) and Hispanic ethnicity reported on death certificates might have been misclassified (*7*), possibly resulting in underestimates of death rates for some groups. Finally, the cause of death for certain persons might have been misclassified. Limited availability of testing for SARS-CoV-2, the virus that causes COVID-19, at the beginning of the COVID-19 pandemic might have resulted in an underestimation of COVID-19–associated deaths.

This report provides an overview of provisional U.S. mortality data for 2020. Provisional death estimates can give researchers and policymakers an early indication of shifts in mortality trends and provide actionable information sooner than the final mortality data that are released approximately 11 months after the end of the data year. These data can guide public health policies and interventions aimed at reducing numbers of deaths that are directly or indirectly associated with the COVID-19 pandemic and among persons most affected, including those who are older, male, or from disproportionately affected racial/ethnic minority groups.

**Summary**

**What is already known about this topic?**

The COVID-19 pandemic caused approximately 375,000 deaths in the United States during 2020.

**What is added by this report?**

The age-adjusted death rate increased by 15.9% in 2020. Overall death rates were highest among non-Hispanic Black persons and non-Hispanic American Indian or Alaska Native persons. COVID-19 was the third leading cause of death, and the COVID-19 death rate was highest among Hispanics.

**What are the implications for public health practice?**

Provisional death estimates provide an early indication of shifts in mortality trends. Timely and actionable data can guide public health policies and interventions for populations experiencing higher numbers of deaths that are directly or indirectly associated with the COVID-19 pandemic.

## Footnotes

*
https://www.cdc.gov/nchs/nvss/deaths.htm

† Based on death records received and processed as of March 21, 2021, for deaths occurring in the United States among U.S. residents. Data included in this analysis include >99% of deaths that occurred in 2020.

§ At the time of analysis, 1.1% of total NVSS deaths and 0.6% of COVID-19 deaths that occurred in the United States were among residents of U.S. territories and foreign countries.
