# *Erratum:* Vol. 70, No. 14

**DOI:** 10.15585/mmwr.mm7024a4

**Published:** 2021-06-18

In the report “Provisional Mortality Data — United States, 2020,” on page 520, the last sentence in the “What is added by this report?” paragraph of the Summary box should have read, “COVID-19 was the third leading cause of death, and the COVID-19 death rate was highest among **non-Hispanic American Indian or Alaska Native persons**.” 

